# Evaluation of automated systems for aminoglycosides and fluoroquinolones susceptibility testing for Carbapenem-resistant *Enterobacteriaceae*

**DOI:** 10.1186/s13756-017-0235-7

**Published:** 2017-08-01

**Authors:** Zhichang Zhao, Fangjun Lan, Maobai Liu, Weiyuan Chen, Liya Huang, Qili Lin, Bin Li

**Affiliations:** 1 Department of Pharmacy, Fujian Medical University Union Hospital, Fuzhou, Fujian 350001 China; 2 Department of Clinical Laboratory, Fujian Medical University Union Hospital, 29 Xinquan Rd, Fuzhou, Fujian 350001 People’s Republic of China; 3 Medical Technology and Engineering College, Fujian Medical University, Fuzhou, 350004 Fujian People’s Republic of China

**Keywords:** Automated identification systems, Carbapenem-resistant *Enterobacteriaceae*, Aminoglycosides, Fluoroquinolones

## Abstract

**Background:**

Automated systems (MicroScan WalkAway 96 Plus, Phoenix 100, and Vitek 2 Compact) are widely used in clinical laboratories nowadays. The aim of this study is to evaluate the performance of these three systems for susceptibility testing of aminoglycosides and fluoroquinolones against Carbapenem-resistant *Enterobacteriaceae* (CRE).

**Methods:**

A total of 75 CRE isolates were used in this study. Quinolone resistance determinants (QRDs) (*qnrA*, *qnrB*, *qnrC*, *qnrD*, *qnrS*, *aac(6′)-Ib-cr*, *oqxAB* and *qepA*) and aminoglycoside resistance determinants (ARDs) (*aac(6′)-Ib*, *armA*, *npmA*, *rmtA*, *rmtB*, *rmtC*, *rmtD* and *rmtE*) of these CRE were screened by PCR. The MICs of aminoglycosides (gentamicin and amikacin) and fluoroquinolones (ciprofloxacin and levofloxacin) to CRE obtained with the automated systems were compared with the reference method (agar dilution method).

**Results:**

Totally, 97.3% (73/75) of CRE harbored QRDs. The *qnr* gene was the most common QRD determinant identified in 68 (96.7%), followed by *aac (6′)-Ib-cr* in 56 (74.7%), *oqxAB* in 23 (30.7%), and *qepA* in 2 (2.7%), respectively. 22.7% (17/75) of CRE harbored ARD determinants. *rmtA*, *rmtB* and *npmA* were identified among these isolates in 6 (8.0%), 6 (8.0%) and 5 (6.7%), respectively. A total of 900 results were obtained in this study. Overall, the total error rate was 9.89%. Twenty-eight very major errors (3.11%), 22 major errors (2.44%) and 39 minor errors (4.33%) were identified against agar dilution method. The very major errors were almost evenly distributed between results for fluoroquinolones (2.89%) and aminoglycosides (3.33%), while the major errors and minor errors were more commonly found in the results of fluoroquinolones (3.11% and 6.44%, respectively) than aminoglycosides (1.78% and 2.22%, respectively).

**Conclusions:**

Our study shows that testing difficulties in susceptibility testing do exist in automated systems. We suggest clinical laboratories using automated systems should consider using a second, independent antimicrobial susceptibility testing method to validate aminoglycosides and fluoroquinolones susceptibility.

## Background

Carbapenem-resistant *Enterobacteriaceae* (CRE) has spread throughout the world nowadays [1–3]. CRE usually show high levels of resistance to many types of antibiotics. Infections caused by CRE will pose a serious threat to patients for the limited therapeutic options. Therefore, CRE infections are always associated with poor outcomes and high mortality rate at the present time [4–6]. The most optimal treatment options for CRE infections have not been well defined. Current treatment options include the use of some older agents either in monotherapy or in combination therapy, such as aminoglycosides and fluoroquinolones [7, 8]. Aminoglycosides and fluoroquinolones are two different important types of antimicrobial agents for the treatment of life-threatening bacterial infections. Aminoglycosides have demonstrated in vitro activity against CRE, and are often used as part of combination regimens [9, 10]. While fluoroquinolones are sometimes used to combat urinary tract infection caused by CRE [11].

The task of clinical laboratories and microbiologists to perform proper antimicrobial susceptibility test and interpretation constitutes an important initial step in implementing appropriate treatments and antibiotic use policies [12]. In this regard, the accuracy of different antimicrobial susceptibility testing methods may allow physicians to prescribe safe and effective antibiotics to treat infections. Automated systems have been used widely in many clinical laboratories for species identification of the pathogens and susceptibility testing. These systems could decrease the labor and save time compared to those for standardized methods. But probable errors reported by automated systems maybe have serious implications for the clinical outcome for patients with multiple drug resistant bacteria infections, such as CRE infections. Unfortunately, there is no information on the accuracy of these systems in detecting the susceptibility to aminoglycosides and fluoroquinolones with CRE currently.

In China, according to the CHINET report, the incidence of CRE has increased remarkably in the last 10 years [13]. In most Chinese hospitals, the susceptilbility of CRE to aminoglycosides and fluoroquinolones are detected using automated systems. Phoenix 100, Vitek-2 Compact and MicroScan WalkAway 96 Plus are the most common automated identification systems currently used in our country. The aim of the present study was to assess the performance of three automated instruments for the susceptibility testing of aminoglycosides and fluoroquinolones against CRE isolates.

## Methods

### Clinical isolates

A total of 75 carbapenem-resistant *Enterobacteriaceae* were included in this study. They were collected at Fujian Medical University Union Hospital in Fuzhou, China, and some were reported previously [14].

### Antimicrobial susceptibility tests

Aminoglycosides (gentamicin and amikacin) and fluoroquinolones (ciprofloxacin and levofloxacin) susceptibility tests were conducted simultaneously following the manufacturers’ instructions using the three different automated systems: MicroScan WalkAway 96 Plus (SIEMENS AG FWB, Germany) with NC 50 cards, Phoenix 100 (Becton, Dickinson and Company, USA) with NMIC/ID-4 panels and Vitek-2 Compact (Bio Mérieux, France) with AST-GN16 cards. All the assays were accomplished in the research laboratory of Fujian Medical University Union Hospital, China. The susceptibility breakpoints of the three commercial systems were interpreted as recommended by the Clinical and Laboratory Standards Institute(CLSI) [15]. Reference MIC values for the tested isolates were determined by agar dilution method with Mueller-Hinton agar according to CLSI guidelines [15]. *E. coli* ATCC25922 was used as quality control strain.

### Detection of quinolone resistance determinants (QRDs) and aminoglycoside resistance determinants (ARDs)

In this study, QRDs refer to plasmid mediated quinolone resistance genes. All the 75 CRE were screened for the presence of QRDs (*qnrA*, *qnrB*, *qnrC*, *qnrD*, *qnrS*, *aac(6′)-Ib-cr*, *oqxAB* and *qepA*) and ARDs (*aac(6′)-Ib*, *armA*, *npmA*, *rmtA*, *rmtB*, *rmtC*, *rmtD* and *rmtE*) with PCR using primers as previously described [16, 17]. PCR products were purified and sequenced on an ABI PRISM 3730 automated sequencer (Applied Biosystems, Foster City, USA).

### Evaluation of concordance among methods

The results were analyzed by using the standard agar dilution method as the reference method. Categorical agreement was defined as a result from any of the three automated system methods that belonged to the same interpretive category (i.e., susceptible, intermediate, or resistant) as that determined with the standard agar microdilution method. Essential agreement was defined as when the same MIC values (within ±1 dilution) were obtained by the automated systems and the reference method. In this study, when both MIC values obtained with automated systems and reference method were under or over the limit concentrations in the automated system, these results were considered to “agreement”. A “very major error” was defined as a result in the susceptible category when an organism was considered resistant by the reference method, but susceptible by the test method. A “major error” was defined when an organism considered susceptible by the reference method was resistant by the test method. A “minor error” was defined when an organism was considered susceptible or resistant either by the reference or the test method, while intermediate by the other method. All tests showing very major errors or major errors were repeated in duplicate by both reference and test methods.

## Results

### Prevalence of the QRDs and ARDs among CRE

In total, 97.3% (73/75) of CRE harbored QRDs. The *qnr* gene was the most common QRD determinant identified in 68 (96.7%), followed by *aac (6′)-Ib-cr* in 56 (74.7%), *oqxAB* in 23 (30.7%), and *qepA* in 2 (2.7%), respectively. Among the 68 *qnr* positive CRE, *qnrA*, *qnrB*, *qnrS* was detected in 64 (94.1%), 17 (25.0%), and 9 (13.2%), respectively. *qnrC* and *qnrD* were not found in this study (Table 1).

**Table 1:** Seventy five carbapenem-resistant *Enterobacteriaceae* isolates containing QDRs and ADRs that were detected in this study

Species (no. of isolates)	QDRs and ADRs expressed	No. of isolates
*Escherichia coli* (18)	*qnrA*	2
	*qnrA* + *oqxB*	1
	*aac(6′)-Ib-cr* + *qnrA*	6
	*aac(6′)-Ib-cr* + *qnrB*	1
	*aac(6′)-Ib-cr* + *NPMA*	2
	*aac(6′)-Ib-cr* + *qnrA* + *RmtB*	2
	*aac(6′)-Ib-cr* + *qnrA* + *NPMA*	3
	*oqxB* + *qnrA*	1
*Enterobacter cloacae* (20)	*aac(6′)-Ib-cr* + *qnrA* + *qnrB*	9
	*aac(6′)-Ib-cr* + *qnrA* + *qnrB* + *qnrS*	1
	*aac(6′)-Ib-cr* + *qnrB* + *qnrS*	1
	*aac(6′)-Ib-cr* + *qnrA* + *oqxB* + *oqxA*+ *RmtA*	1
	*aac(6′)-Ib-cr* + *qnrA*	2
	*aac(6′)-Ib-cr* + *qnrA* + *qepA*	1
	*aac(6′)-Ib-cr*	2
	*qnrA*	2
	*aac(6′)-Ib-cr* + *qnrA* + *qnrS*	1
*Klebsiella pneumoniae* (31)	*qnrA*	3
	*qnrA* + *RmtB*	1
	*qnrA* + *RmtA*	2
	*qnrA* + *qnrB* + *oqxA* + *oqxB*	1
	*qnrA* + *oqxA*+ *RmtA*	1
	*qnrA* + *qnrS* + *oqxB*	1
	*qnrA* + *qnrS* + *oqxA* + *oqxB* + *RmtA*	1
	*aac(6′)-Ib-cr* + *qnrA*	1
	*aac(6′)-Ib-cr* + *qnrA* + *oqxA*	1
	*aac(6′)-Ib-cr* + *qnrA* + *oqxB*	3
	*aac(6′)-Ib-cr* + *qnrA* + *qepA*	1
	*aac(6′)-Ib-cr* + *qnrA* + *qnrS* + *oqxB*	1
	*aac(6′)-Ib-cr* + *qnrA* + *oqxA* + *oqxB*	8
	*aac(6′)-Ib-cr* + *qnrA* + *qnrS*+ *oqxA* + *oqxB*	1
	*aac(6′)-Ib-cr* + *qnrA* + *qnrB* + *oqxB*+ *RmtA*	1
	*aac(6′)-Ib-cr* + *qnrA* + *qnrB* + *qnrS* + *oqxB* + *oqxA* + *RmtA*	1
	*aac(6′)-Ib-cr* + *qnrA* + *oqxB* + *oqxA* + *NPMA*	1
	*aac(6′)-Ib-cr* + *oqxB*+ *oqxA*	1
	*oqxA* + *oqxB*	1
*Klebsiella oxytoca* (4)	*aac(6′)-Ib-cr* + *qnrA*	3
	*aac(6′)-Ib-cr* + *qnrB*+ *qnrS*	1
*Enterobacter aerogenes* (1)	*aac(6′)-Ib-cr* + *qnrB* + *qnrS*	1

22.7% (17/75) of CRE harbored ARD determinants. *rmtA*, *rmtB* and *npmA* were identified among these isolates in 6 (8.0%), 6 (8.0%) and 5 (6.7%), respectively. A*ac(6′)-Ib*, *armA*, *npmA*, *rmtC*, *rmtD* and *rmtE* were not detected in the present study.

### Resistance rates with reference agar dilution method

Generally, based on the agar dilution method, the rates of resistance of CRE to CIP, LEV, GEN and AMK were 53.3% (40/75), 37.3% (28/75), 81.3% (61/75) and 14.7% (11/75), respectively (Table 2). The MIC ranges were 0.125–512 μg/ml for CIP, 0.125–128 μg/ml for LEV, 1–512 μg/ml for GEN and 1–512 μg/ml for AMK. The MIC_50_ was 8 μg/ml for CIP, 2 μg/ml for LEV, 128 μg/ml for GEN and 8 μg/ml for AMK.

**Table 2:** Susceptibility of CRE based on testing methods in this study (*N* = 75)

Testing method^a^	Testing results (%, [no. of strains])											
	CIP			LEV			GEN			AMK		
	S	I	R	S	I	R	S	I	R	S	I	R
ADM	37.3% (28)	9.3% (7)	53.3% (40)	52.0% (39)	10.7% (8)	37.3% (28)	17.3% (13)	1.3% (1)	81.3% (61)	74.7% (56)	10.7% (8)	14.7%(11)
Vitek-2	40.0% (30)	8.0% (6)	52.0% (39)	52.0% (39)	1.3% (1)	46.7% (35)	20.0% (15)	2.7% (2)	77.3% (58)	86.7% (65)	0	13.3%(10)
Phoenix	33.3% (25)	8.0% (6)	53.3% (40)	42.7% (32)	8.0% (6)	48.0% (36)	18.7% (14)	4.0% (3)	77.3% (58)	84.0% (63)	1.3% (1)	14.7% (11)
Microscan	29.3% (22)	8.0% (6)	62.7% (47)	45.3% (34)	6.7% (5)	48.0% (36)	22.7% (17)	4.0% (3)	73.3% (55)	80.0% (60)	5.3% (4)	14.7% (11)

^a^ADM, agar dilution method

### Performance on susceptibility of three automatic systems

A total of 900 results were obtained in this study, corresponding to 300 for Phoenix, 300 for WalkAway and 300 for Vitek. Overall, the total error rate was 9.89% (89/900).

Twenty-eight very major errors (3.11%), 22 major errors (2.44%) and 39 minor errors (4.33%) were identified in this study against agar dilution method (Table 3). The very major errors were almost evenly distributed between results for fluoroquinolones (2.89%) and aminoglycosides (3.33%), while the major errors and minor errors were more commonly found in the results of quinolones (3.11% and 6.44%, respectively) than aminoglycosides (1.78% and 2.22%, respectively). Very major errors were 3.67% of the total number of results for Vitek-2, 2.33% for BD Phonix and 3.33% for WalkAway. Major errors were 2.67% for Vitek-2, 2.00% for BD Phonix and 2.67% for WalkAway. Finally, minor errors were 6.00% for Vitek-2, 4.00% for BD Phonix and 3.00% for WalkAway. As to fluoroquinolones, most of the minor errors were susceptible findings interpreted as in intermediate, and resistance results interpreted as intermediate. The agreement in clinical categories was only 76.0 to 85.4% for CIP and LEV. While as to aminoglycosides, most of the minor errors were resistance results interpreted as intermediate, or susceptible findings interpreted as in intermediate. The agreement in clinical categories was 86.7 to 92.0% for GEN and AMK.

**Table 3:** Summary of the results on CRE obtained with automated methods compared to the results with reference method

Antibiotics	System (concns in mg/l)	% agreement in clinical categories	% essential agreement	No. of errors of different types (%)		
				Very major	Major	Minor
CIP	WalkAway (0.12, 1, 2)	76.0	81.3	2 (0.2)	4 (0.4)	4 (0.4)
	BD Phoenix (0.12, 0.25,0.5, 1,2)	80.0	88.0	2 (0.2)	1 (0.1)	4 (0.4)
	Vitek-2 (0.25, 0.5,1, 2, 4)	85.4	84.0	3 (0.3)	2 (0.2)	7 (0.8)
LEV	Walkaway (4, 8)	78.7	86.7	2 (0.2)	2 (0.2)	3 (0.3)
	BD Phoenix (2, 4, 8)	77.3	84.0	1 (0.1)	2 (0.2)	4 (0.4)
	Vitek-2 (1, 2,4, 8, 16)	81.3	81.3	3 (0.3)	3 (0.3)	7 (0.8)
GEN	Walkaway (4, 8)	86.7	89.3	5 (0.6)	1 (0.1)	2 (0.2)
	BD Phoenix (2, 4, 8)	90.7	90.7	3 (0.3)	2 (0.2)	3 (0.3)
	Vitek-2 (1, 2,4, 8, 16)	89.3	90.7	3 (0.3)	2 (0.2)	3 (0.3)
AMK	Walkaway (8, 16, 32)	92.0	94.7	1 (0.1)	1 (0.1)	0
	BD Phoenix (8, 16, 32)	90.7	89.3	1 (0.1)	1 (0.1)	1 (0.1)
	Vitek-2 (8, 16, 32)	88.0	84.0	2 (0.2)	1 (0.1)	1 (0.1)

## Discussion

CRE have become to one of the most difficult-to-treat pathogens for nosocomial infections due to the lack of treatment options [18, 19]. CRE are usually resistant to many commonly prescribed antimicrobials but may still remain susceptible to one or more antibiotics [20]. Aminoglycosides and fluoroquinolones are sometimes used as a choice to combat CRE infections currently [21, 22]. In China, automated systems are widely used nowadays not only in identification of bacteria, but to perform the antimicrobial susceptibility test as a routine in clinical microbiology laboratory. The present study was conducted to evaluate the performance of automated systems for the susceptibility testing on the two types of antibiotics (aminoglycosides and fluoroquinolones).

In this study, we found that QRD and ARD determinants were widely disseminated among CRE, which were similar to previous studies (Table 1) [23–26]. The resistance rates to fluoroquinolones were about 50% (53.3% to CIP and 37.3% to LEV) based on the reference method, which was lower that in previous larger surveillance data from China [27]. The discrepancy probably due to the detection methods used in the previous study. In this study, the automated systems gave similar resistance rates to CIP, while the resistance rates to LEV were higher obtained with automated systems than that got from reference method. Meanwhile, the resistance rates to aminoglycosides vary with the specific drug (81.7% to GEN and 14.7% to AMK) based on the reference method, which were in line with many previous studies [24, 27]. In this study, the automated systems gave similar resistance rates to both GEN and AMK compared with the reference method.

Previous studies showed that automated systems were thought to be reliable for antibiotic susceptibility test, including aminoglycosides and fluoroquinolones [28, 29]. However, according to the US FDA’s recommendation, the performance of susceptibility tests is considered adequate when the total error rate is <10%, with ≤1.5% of errors being very major errors and ≤3.0% being major errors, and when the overall essential MIC agreement is >90% [30]. Taking these values as a reference, the three automated systems cannot be considered reliable for susceptibility testing of aminoglycosides and fluoroquinolones against CRE in our study for the high very major error rate and overall essential MIC agreement (Table 3).

Our study has several limitations. The study was carried out at a single research laboratory. To reduce biases, the measurements were performed in biological duplicates by blinded researchers on different days. Another limitation was the relatively small sample size. For this reason, we were unable to obtain comprehensive evaluation of the three automated systems for aminoglycoside and fluoroquinolones susceptibility testing for CRE. Lastly, only one reference method was chosen in this study. More accurate methods (such as broth dilution method, E-test) should be used to confirm the AST results.

## Conclusions

This is the first study assessing the accuracy of automated systems in detecting the susceptibility of aminoglycosides and fluoroquinolones to CRE. Our limited study suggests that testing difficulties in susceptibility testing do exist in automated systems. These findings warn us that reporting errors could result in serious implications for the clinical outcome for patients. We suggest clinical laboratories using automated systems should consider using a second, independent antimicrobial susceptibility testing method to validate aminoglycosides and fluoroquinolones susceptibility.

## Abbreviations

ARDs: Aminoglycoside resistance determinant
CRE: Carbapenem resistant *Enterobacteriaceae*
MIC: Minimum inhibitory concentration
QRDs: Quinolone resistance determinants
